# Preparation of the Sodium Alginate-g-(Polyacrylic Acid-co-Allyltrimethylammonium Chloride) Polyampholytic Superabsorbent Polymer and Its Dye Adsorption Property

**DOI:** 10.3390/md16120476

**Published:** 2018-11-29

**Authors:** Shuxian Tang, Ying Zhao, Haitao Wang, Yuqiao Wang, Hexiang Zhu, Yu Chen, Shusen Chen, Shaohua Jin, Ziming Yang, Puwang Li, Sidong Li

**Affiliations:** 1 School of Material Science and Engineering, Beijing Institute of Technology, Beijing 100081, China; tangshuxian@bit.edu.cn (S.T.); yzhaoby@ust.hk (Y.Z.); wangyq22@126.com (Y.W.); bitmsebook@yeah.net (H.Z.); bitchen@126.com (S.C.); jinshaohua@bit.edu.cn (S.J.); 2 Baxter Healthcare Ltd., Shanghai 200031, China; haitao_wang@baxter.com; 3 Agriculture Products Processing Research Institute, Chinese Academy of Tropical Agricultural Sciences, Zhanjiang 524001, China; yangziming2004@163.com (Z.Y.); puwangli@163.com (P.L.); 4 School of Chemistry and Environmental Science, Guangdong Ocean University, Zhanjiang 524088, China; lisidong2210491@163.com

**Keywords:** polyampholytic superabsorbent polymer, pH-dependent, adsorption, Methylene blue

## Abstract

A polyampholytic superabsorbent polymer (PASAP), sodium alginate-g-(polyacrylic acid-co-allyltrimethylammonium chloride) (SA-g-(PAA-co-PTM)), was prepared by free-radical graft copolymerization and characterized. The polymer exhibited pH-dependent swelling behaviors with extremely high swelling ratios, and was saline tolerant. The dye adsorption properties of SA-g-(PAA-co-PTM) were investigated using methylene blue (MB) as a cationic dye model. It was found that its dye adsorption capacity was significantly affected by the TM content in PASAP and pH of dye solution. The dye adsorption kinetics and isotherm obey the pseudo-second-order kinetic model and the Langmuir isotherm model, respectively, and the adsorption process is chemisorption in nature. In addition, SA-g-(PAA-co-PTM) exhibited high MB adsorption capacities in a wide pH range and reusability in at least five adsorption-desorption cycles, indicating its great application potentials as the adsorbent for dye removals from effluents.

## 1. Introduction

Organic dyes are widely used in textile, tannery, paint, paper, and other industries, producing significant amounts of dye-containing wastewater [1]. Most of these dyes contain aromatic structures, and thus are toxic and non-degradable. The discharge of untreated industrial dye effluents imposes threats on the ecology and human health [2]. Consequently, the treatment of dye-containing effluents has been extensively studied, and various treatment methods including coagulation, precipitation, adsorption and filtration [3,4] have been developed. Adsorption is one of the most commonly used treatment methods due to its low-cost, simplicity, high efficiency and environmental friendliness [5], and many adsorbents, such as superabsorbent polymers [6], activated carbon [7], graphene oxide [8], clay [9], and so on, have been used. Superabsorbent polymer (SAP), a three-dimensional cross-linked hydrophilic polymer with the advantages of functionalized groups, high water absorption, environmental safety, and low-cost, has been found to be an ideal adsorbent for dye removal. A variety of novel SAPs have been developed for the removals of different dyes from aqueous solutions [10,11].

The composition of dye-containing wastewater is usually very complex, consisting of a variety of salts, in addition different dyes. The pH of wastewater also varies with its composition, which further complicates its properties. The dye adsorption efficiencies of conventional superabsorbent polymers are significantly affected by the salts and variation of pH of wastewaters [12] and, thus, they are not the optimal choice for the dye removals from complex effluents. Although the SAPs prepared with acrylamide and other non-ionic monomers have been demonstrated with salt-resistances, the structure of acrylamide limits their dye adsorption efficiencies. Polyampholytic superabsorbent polymers (PASAPs) contain both cationic and anionic monomer units and, thus, the degree of dissociation of the functional groups on polymer chain and the electrostatic interactions between ions vary in response to external stimuli, such as pH and ionic strength, resulting in unique swelling properties [13]. Therefore, it can be anticipated that PASAPs possess excellent dye adsorption properties under acidic, basic, neutral and high ionic strength conditions [14,15]. However, the study and application of PASAPs for the dye removals from effluents have been rarely reported.

In the present work, a sodium alginate (SA) based PASAP was synthesized by grafting acrylic acid (AA), the anionic monomer, and allyltrimethylammonium chloride (TM), cationic monomer, onto the SA backbone via free-radical copolymerization. Its dye adsorption behavior was systematically studied using MB as the model dye to reveal the unique dye adsorption property of PASAP.

## 2. Results and Discussion

### 2.1. Structural Characterization of SA-g-(PAA-co-PTM) PASAP

Since the structures of PASAPs can significantly affect their water absorption and dye adsorption properties, it is reasonable to characterize and tune the structures of SA-g-(PAA-co-PTM) PASAP. SA-g-(PAA-co-PTM) PASAPs were prepared with 0%, 2%, 3%, 4%, 5%, and 6% molar percentages of TM in the total monomer that were denoted as TM0%, TM2%, TM3%, TM4%, TM5%, and TM6%, respectively, and structurally characterized to explore their formation mechanism.

#### 2.1.1. FTIR

**Figure 1:**
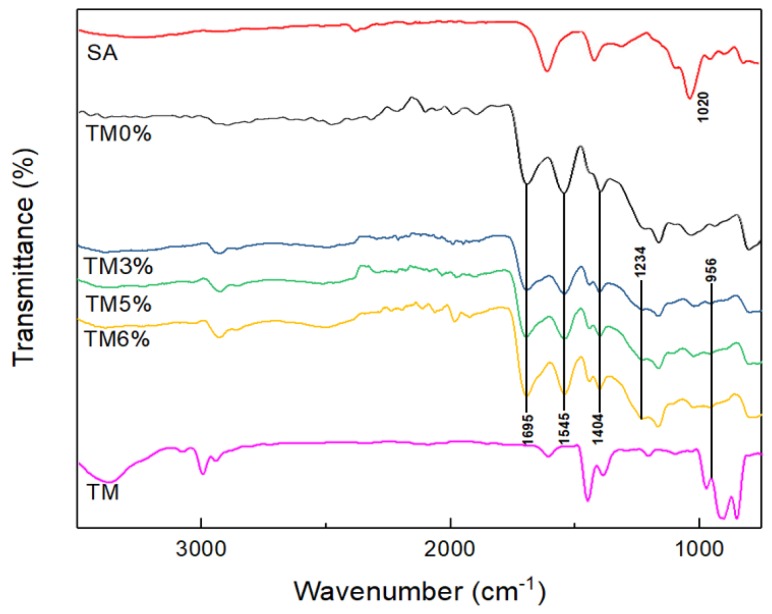
FTIR spectra of sodium alginate, allyltrimethylammonium chloride, and SA-g-(PAA-co-PTM) PASAPs containing 0%, 3%, 5%, and 6% TM (TM0%, TM3%, TM5%, and TM6%).

Figure 1 shows the FTIR spectra of SA, TM and SA-g-(PAA-co-PTM) polymers containing different TM contents. The stretching vibration of C–OH at 1020 cm^−1^ in SA was weakened after the copolymerization reaction. Both stretching vibration peaks of C=C at 3018 cm^−1^ and =C–H at 1635 cm^−1^ in the monomers were weakened, and a saturated skeletal vibration and C–H rocking vibration peaks appeared at 1165 cm^−1^ and 800 cm^−1^, respectively, in the PASAPs, suggesting that the polymerization reaction broke the C=C bonds in the monomers and long carbon chains were formed. The absorption peak at 1695 cm^−1^ was ascribed to the C=O in –COOH of PAA, and those at 1545 cm^−1^ and 1404 cm^−1^ were attributed to the asymmetric and symmetric stretching vibrations of –COO^−^ in PAA, respectively. The absorption peak of the polymers at 1234 cm^−1^ was due to the stretching vibration of C–N. The bending vibration peak of –N^+^(CH_3_)_3_ in PTM and TM was found at 956 cm^−1^ [16]. These results suggest that PAA and PTM chains have been successfully grafted onto the backbone of SA, forming SA-g-(PAA-co-PTM) PASAPs.

#### 2.1.2. XRD Analysis

**Figure 2:**
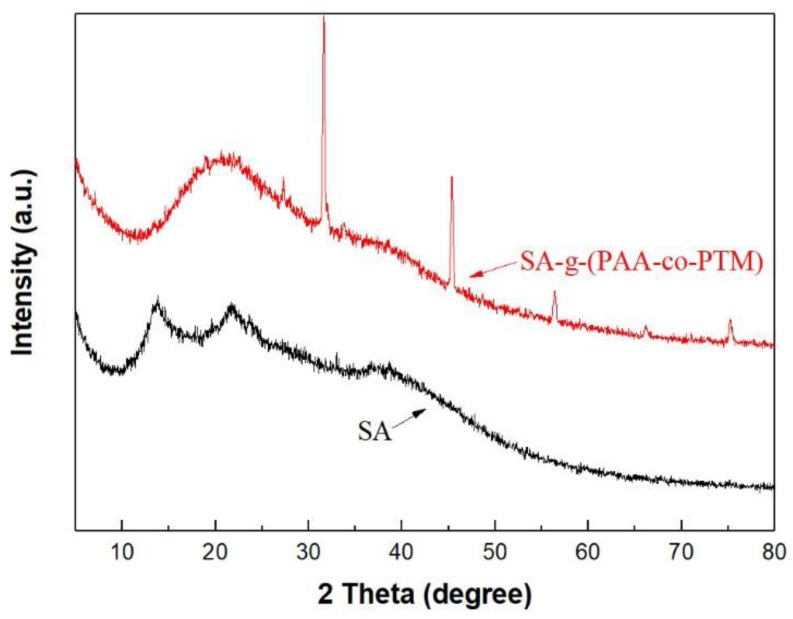
XRD spectra of sodium alginate, and SA-g-(PAA-co-PTM) PASAP containing 2% TM (TM2%).

SA exhibited two dispersion peaks at 2*θ* = 13.73° and 21.71° (Figure 2), indicating that it was amorphous. In contrast, SA-g-(PAA-co-PTM) PASAP showed four sharp crystalline peaks at 27.30°, 31.63°, 45.37°, and 75.22°, and two microcrystalline peaks at 56.41° and 66.17°, suggesting that the graft polymerization and cross-linking produced crystalline due to the reduced molecular distance. The dispersion peak of SA-g-(PAA-co-PTM) was found between the two dispersion peaks of SA, and was obviously wider than those of SA, indicating that the intermolecular forces were increased because of the introduction of carboxyl and quaternary ammonium groups. These results further confirmed the graft polymerization between SA and the monomers.

#### 2.1.3. Morphologies of SA-g-(PAA-co-PTM) PASAP

**Figure 3:**
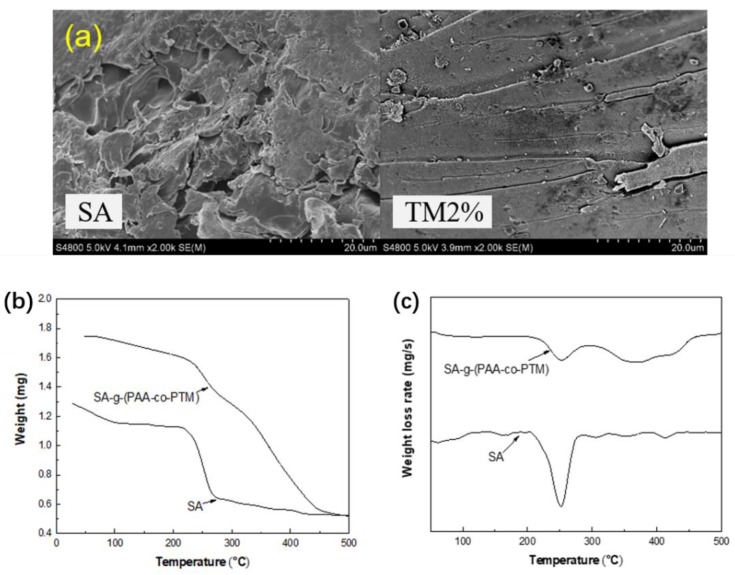
(**a**) SEM images, (**b**) TGA curves, and (**c**) DTG curves of SA and SA-g-(PAA-co-PTM) PASAP containing 2% TM (TM2%).

Figure 3a shows the SEM images (×2000) of SA and SA-g-(PAA-co-PTM) PASAP. SA exhibited a fragment-like loose surface, while the surface of PASAP was relatively continuous and coarse.

#### 2.1.4. TGA Analysis

The thermal properties of SA and SA-g-(PAA-co-PTM) were investigated by TGA and DTG. As shown in Figure 3b,c, the weight loss of SA mainly occurred in the temperature range of 200–283 °C. The maximum decomposition temperature was found at 251 °C with the weight loss of 40.02%, which was due to the decomposition of SA molecule. SA-g-(PAA-co-PTM) exhibited three weight losses with the parameters shown in Table 1. The first two weight losses were respectively due to the thermal degradation of SA, and the decomposition of PAA and PTM functional groups in the branched chains that released CO_2_. The polymer backbones were further decomposed and some binding sites of polymer chain were also decomposed as temperature further increased, resulting in the third weight loss. The temperature for the initial degradation of SA-g-(PAA-co-PTM), the temperature for the maximum weight loss rate, and the temperatures at which the sample lost 5% and 10% of its weight were significantly higher than those for the corresponding weight losses of SA, suggesting the better thermal stability of SA-g-(PAA-co-PTM) due to its cross-linked network.

**Figure 4:**
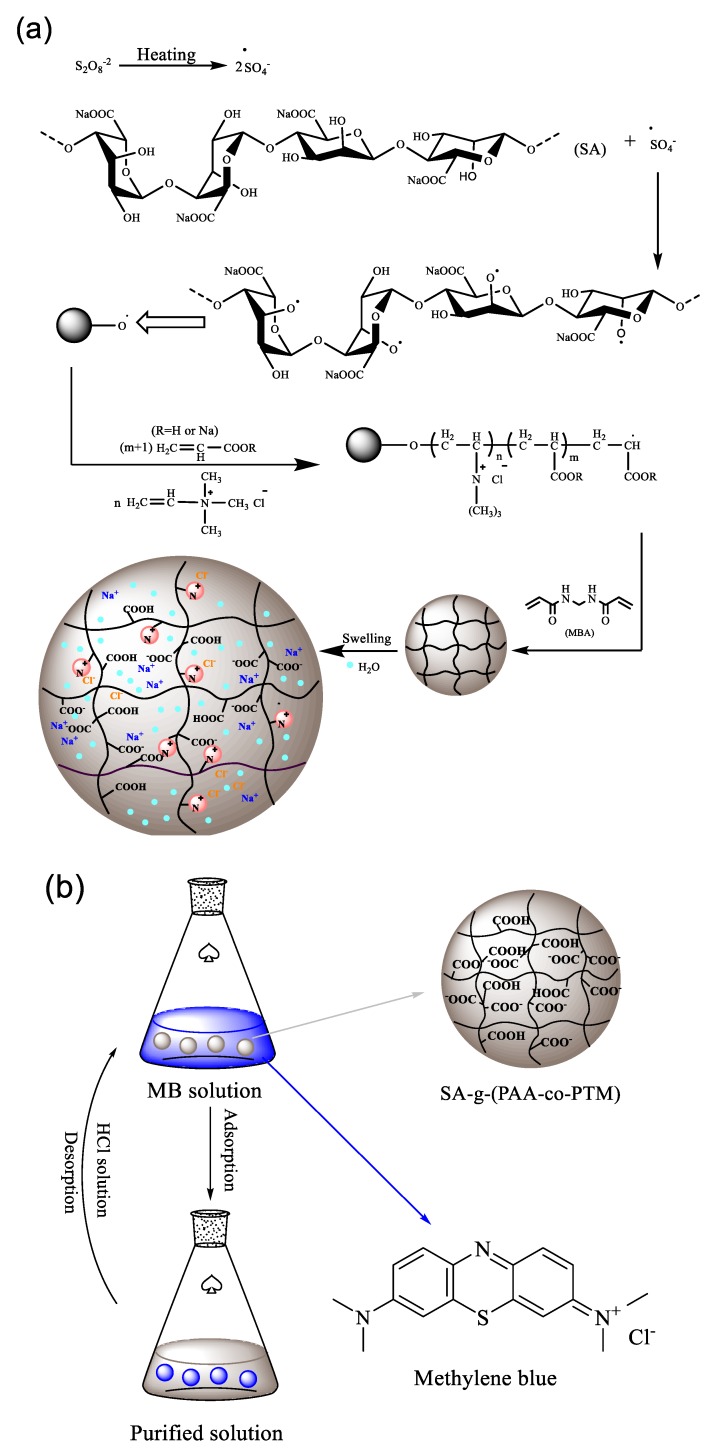
Proposed reaction and swelling mechanism of SA-g-(PAA-co-PTM) PASAP and schematic diagram for MB adsorption mechanism. (**a**) Reaction and swelling mechanism; (**b**) schematic diagram for MB adsorption process.

Based on these results, the formation mechanism of SA-g-(PAA-co-PTM) PASAP is proposed as shown in Figure 4a. Free radicals are generated from the decomposition of initiator and transferred onto SA to initiate the polymerization of AA and TM. Both monomers are then grafted onto the SA backbone. Polymer chains are then cross-linked to form a three-dimensional network as the cross-linker added. Figure 4b shows the proposed adsorption process of MB.

### 2.2. Optimization of Synthesis Conditions

**Figure 5:**
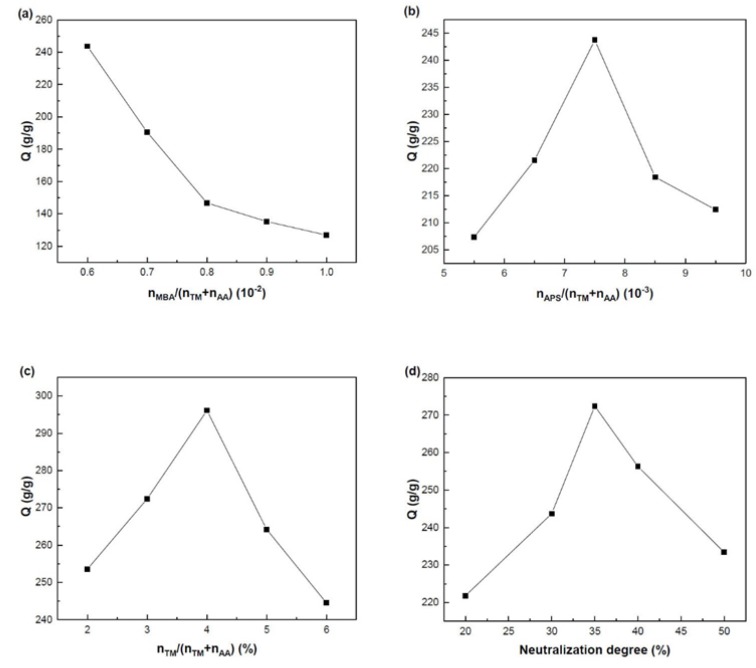
Effects of synthesis conditions on the swelling properties of SA-g-(PAA-co-PTM) PASAP. (**a**) amount of cross-linker; (**b**) amount of initiator; (**c**) monomer ratio; (**d**) neutralization degree of AA.

The water absorption of SAPs varies with their synthesis conditions and structures, which further affects their application. Here, the effects of synthesis conditions on the swelling properties of SA-g-(PAA-co-PTM) PASAP were investigated. The results are shown in Figure 5.

#### 2.2.1. Effects of the Amount of Cross-Linker

Cross-linker is used to form the three-dimension structure of PASAP, which can affect the tightness of the structure and thus the water absorption capacity. As shown in Figure 5a for the effect of amount of cross-linker MBA on the swelling ratio of PASAP, the swelling ratio of PASAP decreased with the increase of MBA amount ratio to the total monomer amount in the range of 0.6 × 10^−2^–1.0 × 10^−2^. It can be explained that a higher amount of cross-linker produced a tenser structure of PASAP. The average molecular weight between cross-linking points became too low for the polymer swelling. As shown in Flory equation (Equation (1)), the equilibrium swelling ratio *q_m_* decreases with the increase of the cross-linking density *v_e_/V*_0_:

(1) qm53≅[(i/2vuS*1/2)2+(1/2−X1)/v1]/(ve⁄V0)

#### 2.2.2. Effects of the Amount of Initiator

As shown in Figure 5b, the swelling ratio of PASAP varied significantly with the increase of the amount of initiator. The concentration of macroradicals in the main chain of SA was low at the n_APS_/(n_TM_ + n_AA_) less than 7.5 × 10^−3^ due to the small amount of initiator, and consequently, the graft polymerization reaction rate was also very low. With more initiator added into the system, the three-dimensional network was easily formed, which further increased the water absorption capacity. However, in the free radical copolymerization, the mean kinetic chain length decreased with the increase of the concentration of initiator [17]. The termination reaction of radicals was enhanced via bimolecular collision as n_APS_/(n_TM_ + n_AA_) increased to over 7.5 × 10^−3^ and the molecular weight of the branched chains between cross-linking points was decreased [18]. Therefore, the network was partially formed, and the water absorption capacity was decreased. The maximum water absorption capacity was achieved at n_APS_/(n_TM_ + n_AA_) = 7.5 × 10^−3^.

#### 2.2.3. Effects of the Molar Ratio between the Monomers

As shown in Figure 5c, the relative content of TM exhibited significant effects on the water absorption capacity of PASAP. At molar ratios n_TM_/(n_TM_ + n_AA_) lower than 4%, increasing the amount of TM containing –N^+^(CH_3_)_3_ enhanced the electrostatic interactions between cations and anions of the polymer chains and, thus, increased the degree of cross-linking. The structure of the dimensional network was more complete and, thus, the water absorption capacity was increased. However, at the n_TM_/(n_TM_ + n_AA_) higher than 4%, the excessive TM caused higher cross-linking degrees of the network, which decreased the water absorption capacity. In addition, the carboxyl group of AA is more hydrophilic, which is conducive to water absorption [19]. The steric effect of TM is significant. Therefore, increasing the amount of TM might further reduce the reaction activity of monomers. The water absorption capacity was decreased due to the incomplete network.

#### 2.2.4. Effects of the Neutralization Degree of AA

Figure 5d shows the effects of neutralization degree of AA on the water absorption capacity of the PASAP. The maximum water absorption capacity was found at the neutralization degree of 35%. It can be explained that the dissociation of AA was weak at lower neutralization degree and the concentration of –COO^−^ was low, resulting in weak repulsive forces between anions. In addition, the concentration of Na^+^ was lower at lower neutralization degrees, and thus the osmotic pressure between the inside and outside of the network was low. Therefore, the water absorption capacity of PASAP increased with the increase of neutralization degree of AA. However, the reactivity of AA with higher neutralization degrees was low, and the strengthened repulsive forces between –COO^−^ groups resulted in a less dense network. Consequently, the network of polymer tended to be incomplete, which was inconducive to water absorption.

Based on these results, the optimal synthesis conditions were determined to be n_MBA_/(n_AA_ + n_TM_) = 0.6 × 10^−2^, n_APS_/(n_AA_ + n_TM_) = 7.5 × 10^−3^, n_TM_/(n_AA_ + n_TM_) = 4% and neutralization degree of AA 35%. The water absorption of the PASAP prepared under the optimal synthesis conditions was measured to be 296.1 g/g.

### 2.3. Effects of Solution Property on the Swelling Properties of SA-g-(PAA-co-PTM) PASAP

#### 2.3.1. pH

**Figure 6:**
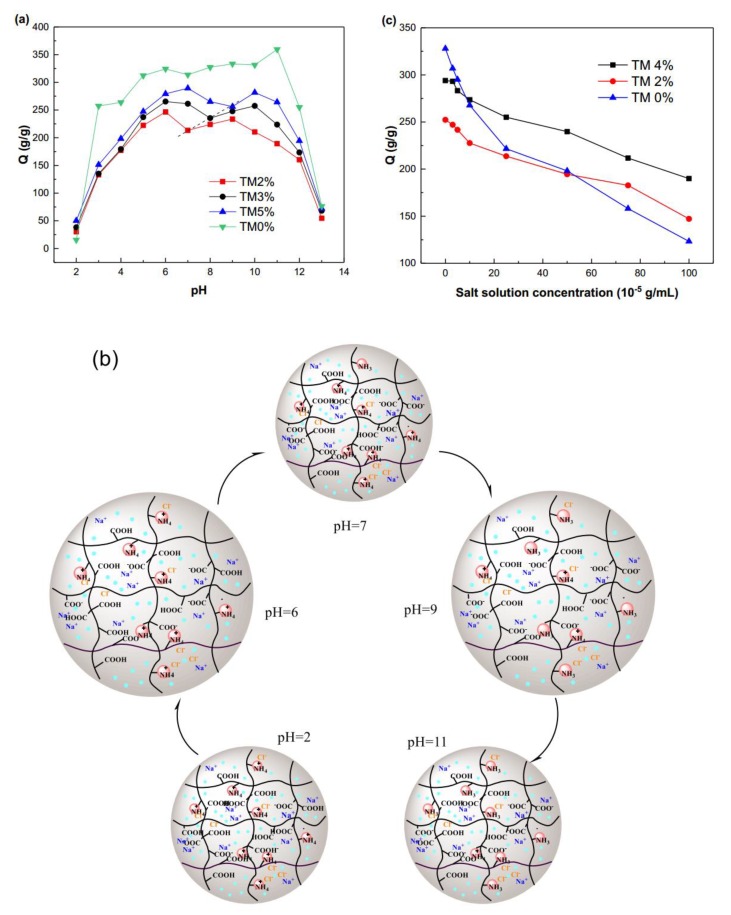
Swelling behaviors of SA-g-(PAA-co-PTM) PASAP. (**a**) Effects of solution pH, and (**b**) the proposed swelling mechanism in solutions of different pHs; (**c**) Effects of salt solution concentration.

The water absorption capacities of the SA-g-(PAA-co-PTM) PASAP prepared under different conditions in the pH range of 2–13 were measured. As shown in Figure 6a, the polyanionic SAP (TM0%) exhibited only one single swelling peak under basic conditions. In contrast, all PASAPs showed two swelling peaks in the test pH range and there was no significant difference upon the peak values. The minimum swelling ratio was observed at the isoelectric point (IEP) between the two peaks, where the numbers of cationic and anionic groups were equal [20]. The unique swelling behaviors of SA-g-(PAA-co-PTM) PASAP are mainly attributed to the electrostatic interactions between the ions on the polymer chain. Figure 6b shows the pH responsive swelling mechanism of SA-g-(PAA-co-PTM) PASAP.

A large number of Cl^−^ ions gathered around –N^+^(CH_3_)_3_ at pHs lower than 3, resulting in a screening effect which weakened the repulsive forces between –N^+^(CH_3_)_3_ ions. Meanwhile, the –COOH group was slightly dissociated under strong acid conditions, and thus the repulsion between anions –COO^−^ was negligible, resulting in lower water absorption capacities at low pHs. The concentration and screening effect of Cl^−^ decreased with the increase of pH, and the repulsive forces between –N^+^(CH_3_)_3_ were strengthened accordingly. Eventually, the swelling ratio peaked under weak acid conditions, and further increasing pH dramatically decreased the swelling ratio to the lowest point. It can be explained that the ionization degree of –COOH increased with the increase of pH, and the number of –COO^−^ anions became approximately equal to that of –N^+^(CH_3_)_3_ cations at the IEP. The strong electrostatic forces between the cation and anion significantly increased the cross-link density of polymer, resulting in the low swelling ratio. As the pH increased to over the IEP, more –COOH groups were dissociated. The large amount of –COO^−^ groups repulsed each other, and the repulsive force increased with the increase of pH, resulting in the peak swelling ratio of PASAP under weak basic conditions. However, as pH exceeded 10, the screening effect of Na^+^ counter cations on –COO^−^ increased with the increase of pH as large amounts of NaOH added, which weakened the repulsion forces between anions. Eventually, the network of the swollen polymer collapsed and the water absorption capacity decreased sharply under the strong salt screening effect.

The on-off switchable swelling behavior of the PASAP in response to pH endows it pH responsive water absorption. The maximum water absorption capacity was obtained under both acidic and basic conditions, with the lowest water absorption at the IEP of PASAP [21]. In contrast, the swelling of conventional polyanion SAPs is off under both strong acidic and basic conditions [22]. Therefore, the SA-g-(PAA-co-PTM) PASAP can be potentially used for dye separation from wastewater via its pH responsive swelling properties. It swells to absorb dye at high or low pHs, and shrinks at the IEP to separate the dye. 

The IEP of SA-g-(PAA-co-PTM) PASAP varies with its TM content, increasing with the increase of TM content (Figure 6a). It can be explained that the ionization degree of -COOH must be increased to equate the –N^+^(CH_3_)_3_ cations and –COO^−^ anions on the polymer chain of SA-g-(PAA-co-PTM) PASAP containing a higher amount of TM. The tunable IEP of SA-g-(PAA-co-PTM) PASAP significantly promotes its application in the dye removal from wastewaters with different properties. PASAP with suitable isoelectric points can be used as the absorbents to simplify the dye separation and improve dye absorption efficiency.

#### 2.3.2. Ionic Strength

The water absorption capacity of SAP is very sensitive to the ionic strength of absorption solution. Figure 6c shows the water absorption capacities of SA-g-(PAA-co-PTM) PASAP containing different amounts of TM in the salt solutions of different concentrations. The swelling ratio of SA-g-PAA SAP decreased rapidly with the increase of salt concentration, while that of SA-g-(PAA-co-PTM) PASAP decreased gradually with the increase of salt concentration at low levels. In addition, the swelling ratio decreased more slowly for the PASAP with higher TM contents. These results indicated that the SA-g-(PAA-co-PTM) PASAP was highly salt-tolerant, which is conducive to the dye removal from wastewaters containing high amounts of salts [23]. 

### 2.4. Dye Adsorption Properties

The unique swelling properties of SA-g-(PAA-co-PTM) PASAP make it a suitable absorbent for the dye removals under various conditions. Here, its dye adsorption properties were investigated using methylene blue (MB) as a model dye.

#### 2.4.1. Effects of TM Content

**Figure 7:**
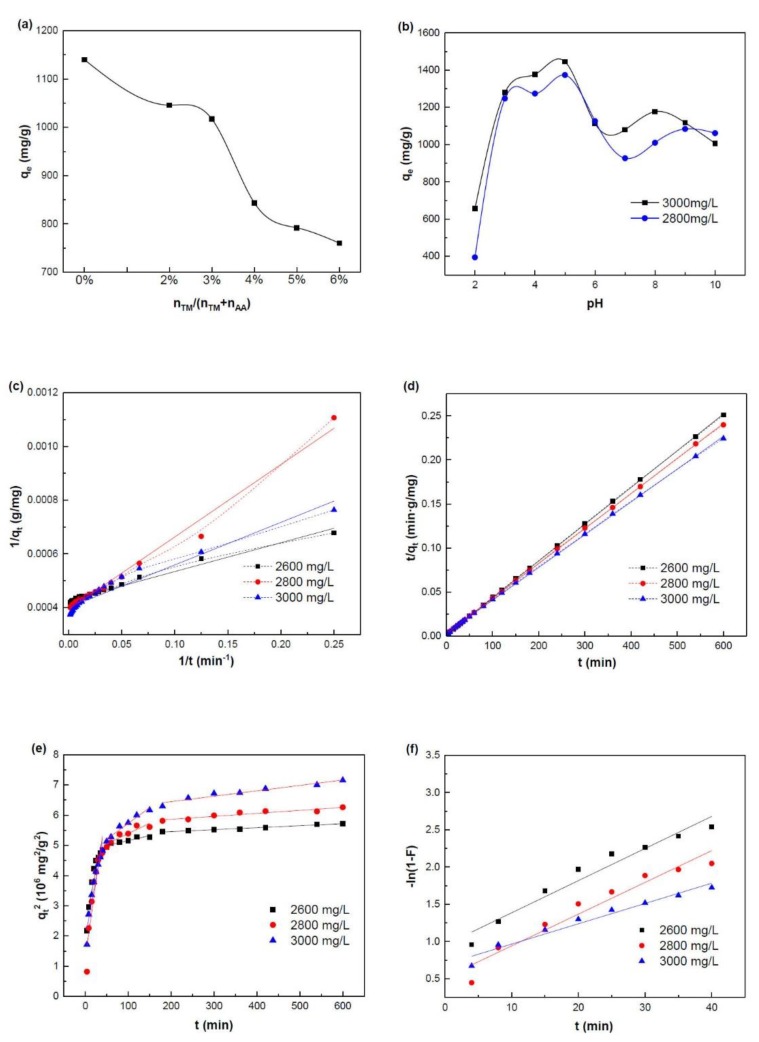
MB adsorption properties and kinetics of SA-g-(PAA-co-PTM) PASAP. (**a**) Effects of the amount of TM on the MB adsorption property; (**b**) Effects of solution pH on the MB adsorption property; (**c**) Pseudo-first-order model; (**d**) Pseudo-second-order model; (**e**) Weber Morris model; and (**f**) Boyd model. The PASAP used in (**b**)–(**f**) was TM2%.

As shown in Figure 7a, the MB adsorption capacity of SA-g-(PAA-co-PTM) PASAP decreased from 1139.9 to 760.3 mg/g as the relative TM content increased from 0% to 6%. The low adsorption capacities of the PASAPs with higher TM contents can be explained by their weaker electrostatic interactions with MB. Increasing TM content weakened the interactions between the –COO^−^ of PASAP and the cationic groups in MB and, thus, decreased the dye adsorption capacity. However, the MB adsorption capacity decreased slowly with the increase of TM content from 0% to 3%. Therefore, the slight variations of TM content only affect the dye adsorption capacity slightly of SA-g-(PAA-co-PTM) PASAP, despite the significant influences on its swelling properties and pH-sensitivity.

#### 2.4.2. Effects of Solution pH

The MB adsorption capacities of SA-g-(PAA-co-PTM) PASAP (TM2%) at different pHs in the range 2 to 10 were measured. As shown in Figure 7b, the adsorption capacity exhibited similar change trends with the variation of pH at the initial MB concentrations of 3000 and 2800 mg/L, increasing dramatically with the increase of pH from 2 to 5, and reaching the maximum (1445.4 mg/g) at pH 5, comparable to the highest MB adsorption capacity of conventional adsorbents reported so far (Table 2). The variation of adsorption capacity was attributed to the change of electrostatic interaction between the –COO^−^ on PASAP and the cationic groups of MB with pH. The concentration of –COO^−^ was low at low pHs and, thus, the electrostatic interaction between the PASAP and MB was weak, resulting in low dye adsorption capacities at low pHs. The concentration of –COO^−^ increased with the increase of pH, and correspondingly, the electrostatic interaction was increased, resulting in higher adsorption capacities. The MB adsorption capacity of the SA-g-(PAA-co-PTM) PASAP was measured to be 1250–1445 mg/g, indicating that it was suitable for cationic dye removals in a wide pH range. Further increasing pH to 6–7, near the IEP of SA-g-(PAA-co-PTM) PASAP, resulted in declined MB adsorption capacities, yet at a relatively high level, due to the unique swelling behavior of the polyampholyte. It can be explained that the cross-link density of SA-g-(PAA-co-PTM) PASAP became high at the IEP, which limited the swelling and expansion of the polymer and, thus, lowered the number of dye adsorption sites on the polymer surface, resulting in the relatively low adsorption capacities. The MB adsorption capacity remained at the level of ~1100 mg/g with slight fluctuations in the pH range of 8 to 10.

In all, SA-g-(PAA-co-PTM) PASAP exhibited higher MB adsorption capacities, even at its IEP, as compared with those of conventional adsorbents. In addition, the shrinking behavior of the PASAP can be used to separate dyes from wastewater.

#### 2.4.3. Adsorption Kinetics

The MB adsorption kinetics of SA-g-(PAA-co-PTM) PASAP was determined using sample TM2% and the MB solutions with the initial concentrations of 2600 mg/L, 2800 mg/L, and 3000 mg/L, respectively.

To determine the controlling steps of the MB adsorption on SA-g-(PAA-co-PTM) PASAP, the experimental data were fitted with four models including pseudo-first-order model (Equation (2)), pseudo-second-order (Equation (3)) kinetic model, Weber Morris model (intra-particle diffusion model) (Equation (4)), and Boyd model (film diffusion model) (Equation (5)).

The pseudo-first-order and pseudo-second-order kinetic models for the MB adsorption of SA-g-(PAA-co-PTM) PASAP in an aqueous phase can be expressed as follows:

(2) $$\frac{1}{q_{t}}=\left(\frac{k_{1}}{q_{e1}}\right)\left(\frac{1}{t}\right)+\frac{1}{q_{e1}}$$

(3) $$\frac{t}{q_{t}}=\frac{1}{k_{2}\;q_{e2}^{2}}+\frac{1}{q_{e2}}\times t$$

where *q_t_* (mg/g) is the amount of dye absorbed at time *t*, *q_e*1*_* (mg/g) and *q_e*2*_* (mg/g) are the equilibrium adsorptions, and *k_*1*_* (min^−1^) and *k_*2*_* (g/(mg min)) are the rate constants of the pseudo-first-order and pseudo-second-order models, respectively.

The fitting linear plots and kinetic parameters calculated from the pseudo-first-order and pseudo-second-order models are depicted and summarized in Figure 7c,d, and Table 3a. The coefficients of determination of both fitting curves, $R_{1}^{2}$ and $R_{2}^{2}$, are high, yet the pseudo-second-order model fits the adsorption process better. In addition, the equilibrium uptake of MB calculated by the pseudo-second-order model is more consistent with the experimental data. These results demonstrate that the MB adsorption on SA-g-(PAA-co-PTM) PASAP followed the pseudo-second-order kinetics, and thus the chemisorption involving valence forces through sharing or exchange of electrons between the adsorbent and adsorbate might be the rate-determining step [28]. The cationic MB dye adsorbed on SA-g-(PAA-co-PTM) PASAP via the electrostatic interaction. With the proceeding of adsorption, the synergetic effects of the electrostatic repulsion between MB cations, and steric hindrance prevented more dye molecules to adsorb on the PASAP, reaching the adsorption equilibrium eventually.

The diffusion-based models, Weber Morris model (intra-particle diffusion model) (Equation (4)) and Boyd model (film diffusion model) (Equation (5)), were then fitted to determine the diffusion mechanism of MB adsorption on SA-g-(PAA-co-PTM) PASAP:

(4) $$q_{t}^{2}=k_{pi}t+C_{i}$$

where *k_pi_* (mg^2^/(g^2^·min)) is intra-particle diffusion rate constant, and the constant *C_i_* is proportional to the boundary layer *i*.

(5) $$-\ln\left(1-F\right)=k_{fd}t$$

where *F = q_t_*/*q_e_*, and *k_fd_* (min^−1^) is the adsorption rate constant of Boyd model.

In general, a dye adsorption process involves three stages: (1) the initial diffusion of dye molecules from the aqueous phase to the exterior surface of adsorbent; (2) the diffusion of the dye molecules into the interior of adsorbent; and (3) the uptake of dye. The slowest step, usually either the film diffusion or intra-particle diffusion, controls the overall adsorption rate.

The fitting plots and parameters of the diffusion-based models are shown in Figure 7e,f and Table 3a. The Weber-Morris plot consists of three linear segments, corresponding to the three stages of MB adsorption on the PASAP (Figure 7e), among which the intra-particle diffusion shown as the second segment is the rate-controlling step. The plots do not pass through the origin, indicating that the intra-particle diffusion is not the only controlling step and other mechanisms may be involved in the adsorption process as well. Only the data of the first 40 min (film diffusion) fit the Boyd model, and the plots do not pass the origin either, indicating that film diffusion is also involved in the adsorption process, but not exclusively [29]. Based on these findings, it can be concluded that both film diffusion and intra-particle diffusion control the MB adsorption, and the adsorption process can be depicted as: (1) MB molecules diffuse from the solution to the exterior surface of SA-g-(PAA-co-PTM) PASAP as it swells; (2) the MB molecules diffuse into the internal SA-g-(PAA-co-PTM) network due to the electrostatic interaction with the –COO^−^ of the PASAP, and (3) the MB molecules adsorb around the –COO^−^. The number of available adsorption sites and the concentration of MB in solution phase are gradually decreased, the diffusion becomes difficult, and eventually, the adsorption reaches the equilibrium state [30].

#### 2.4.4. Adsorption Isotherm

Adsorption isotherm can be used to describe the adsorption behavior, capacity and affinity of an adsorption system. The MB adsorption isotherm was fitted with three isotherm models including the Langmuir isotherm model, the Freundlich isotherm model, and the Dubinin-Radushkevich (D-R) model.

Assuming that the adsorption is monolayer, and the adsorbent surface is homogeneous, the Langmuir isotherm model can be expressed as Equation (6):

(6) $$\frac{c_{e}}{q_{e}}=\frac{c_{e}}{q_{m}}+\frac{1}{K_{L}q_{m}}$$

The Freundlich isotherm model describing the multilayer adsorption on heterogeneous surfaces with different adsorption energies can be expressed as Equation (7):

(7) $$\ln q_{e}=b_{F}\ln c_{e}+\ln K_{F}$$

The physical and chemical adsorptions are usually distinguished by the D-R model that can be expressed as Equation (8):

(8) $$\ln q_{e}=K\varepsilon^{2}+\ln q_{DR}$$

where *q_e_* (mg/g) is the equilibrium adsorption capacity, *c_e_* (mg/L) is the equilibrium dye concentration, *K_L_* (L/mg) is a adsorption affinity constant, *K_F_* and *b_F_* are the adsorption capacity and intensity constants, *q_m_* and *q_DR_* (mg/g) are the maximum adsorption capacities in the Langmuir and D-R models, respectively, *K* (mol^2^/kJ^2^) is the D-R constant, and ε is the Polanyi potential given by Equation (9):

(9) $$\varepsilon =RT\;\ln\left(1+1/c_{e}\right)$$

where *R* is the gas constant (8.314 J/(mol K)), and *T* is the temperature (K).

The D-R model can give the mean adsorption energy by Equation (10):

(10) $$E={\left(-2K\right)}^{-1/2}$$

where *E* is the mean adsorption energy, and *K* (mol^2^/kJ^2^) is the D-R constant.

**Figure 8:**
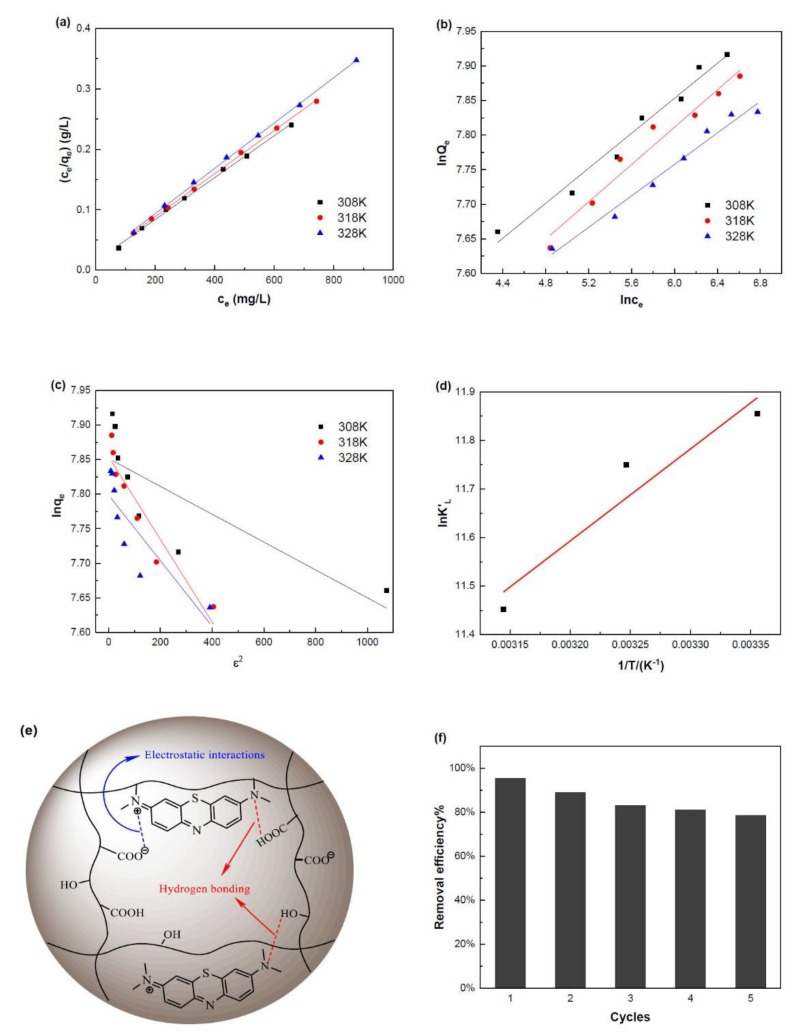
Adsorption isotherms, thermodynamics, mechanism and reusability of MB on SA-g-(PAA-co-PTM) PASAP (TM2%). (**a**) Langmuir model; (**b**) Freundlich model; (**c**) Dubinin-Radushkevich model; (**d**) thermodynamics; (**e**) mechanism; and (**f**) reusability.

The fitting curves and parameters are shown in Figure 8a–c and Table 3b. The linear regression coefficient of determination (*R^2^*) of Langmuir model is higher than those of other two models, indicating that the Langmuir isotherm model is more suitable to describe the adsorption equilibrium of MB on SA-g-(PAA-co-PTM) PASAP, and the adsorption is monolayer on a homogenous surface.

The nature of adsorption, i.e., physisorption or chemisorption, can be predicted with the mean value of adsorption energy (*E*) that is defined as the free energy change of one-mole ions as transferred from the infinity in a solution to the surface of a solid. The adsorptions with *E* lower than 8 kJ/mol, between 8 and 16 kJ/mol, and higher than 20 kJ/mol are considered as physisorption, ion exchange, and chemisorption, respectively [31]. The *E* of the MB adsorption on SA-g-(PAA-co-PTM) PASAP was in the range from 26.78 kJ/mol to 49.81 kJ/mol, suggesting that the adsorption was dominated by chemisorption. The chemisorption of MB on SA-g-(PAA-co-PTM) PASAP takes place via the electrostatic interaction between MB cation and carboxylic anion, and the hydrogen bonding formed between the nitrogen of MB and the hydroxyl and unionized carboxyl of SA-g-(PAA-co-PTM). The electrostatic interaction between MB cation and carboxylic anion is stronger and, thus, dominates the adsorption [32]. The adsorption mechanism is proposed as shown in Figure 8e.

The adsorption affinity can be evaluated with the separation factor (*R_L_*) calculated by Equation (11):

(11) $$R_{L}=1/\left(1+K_{L}c_{0}\right)$$

where *K_L_* is the Langmuir equilibrium constant and *c*_0_ (mg/L) is the initial dye concentration. It is unfavorable if *R_L_* > 1, linear if *R_L_* = 1, favorable if 0 < *R_L_* < 1, and irreversible if *R_L_* = 0. A lower *R_L_* indicates the stronger affinity between the adsorbate and adsorbent [33]. The *R_L_* of the MB adsorption on SA-g-(PAA-co-PTM) PASAP was determined to be 0.0208–0.0238 (Table 3b), implying the strong adsorption affinity.

#### 2.4.5. Thermodynamics of Adsorption 

The thermodynamics of adsorption was investigated to determine the spontaneity of the MB adsorption on SA-g-(PAA-co-PTM) PASAP. The parameters including enthalpy change (Δ*H*°), entropy change (Δ*S*°), and free energy change (Δ*G*°) of the adsorption were calculated with experiment data by the Van’t Hoff equation (Equation (12)):

(12) $$\ln K_{L}=-\Delta H{}^{\circ}/RT+\left(\Delta S{}^{\circ}\right)/R$$

where *K_L_* is the Langmuir equilibrium constant, *R* is the universal gas constant (8.314 J/mol K), and *T* is the absolute temperature (K). Δ*H*° and Δ*S*° can be calculated from the slope and intercept of the plot of ln*K_L_* vs. 1/*T* (Figure 8d and Table 4), respectively.

The negative Δ*H*° indicates that the adsorption of MB on SA-g-(PAA-co-PTM) PASAP is exothermic and the positive Δ*S*° suggests that the randomness increases as the adsorption proceeded (Table 4). Δ*G*° can be obtained from Equation (13):

(13) ΔG°=ΔH°−TΔS°

All of three Δ*G*° are negative, suggesting that the MB adsorption is a spontaneous process, whereby no input energy outside of the system required. The absolute Δ*G*° value increases with the increase of temperature, indicating that high temperatures can promote the MB adsorption on SA-g-(PAA-co-PTM) PASAP. 

In general, the Δ*G*° of physisorption is in the range from 0 to −20 kJ/mol, and that of chemisorption is from −80 to −400 kJ/mol [34]. The Δ*G*° of MB adsorption on SA-g-(PAA-co-PTM) PASAP was found to be in the range from −24.91 to −23.75 kJ/mol, indicating that the adsorption was chemisorption in nature, and enhanced by the physisorption, consistent with the conclusion drawn from the isotherm analyses.

#### 2.4.6. Reusability

Reusability is an important parameter to evaluate the practical application potential of an adsorbent. The MB adsorbed on SA-g-(PAA-co-PTM) PASAP can be desorbed in diluted HCl solution because the –COO^−^ is converted into –COOH in acidic solutions, and correspondingly, the electrostatic interactions between SA-g-(PAA-co-PTM) and MB are weakened. The reusability of SA-g-(PAA-co-PTM) PASAP was evaluated with the adsorption-desorption cycles of MB. The MB removal efficiency was 95.40% at the first cycle and retained at a relatively high level at the fifth cycle (Figure 8f) [35], indicating that SA-g-(PAA-co-PTM) PASAP was reusable for MB removal.

## 3. Materials and Methods 

### 3.1. Materials

Sodium alginate (SA) and allyltrimethylammonium chloride (TM, AR grade) were purchased from Aladdin Industrial Co., Ltd (Shanghai, China). The *M_w_* and *G/M* ratio of SA were 120 kDa and 35/65, respectively. Acrylic acid (AA, AR grade) was purchased from Tianjin Fuchen Chemical Reagents Factory (Tianjin, China). A mmonium persulfate (APS, AR grade) and *N, N’*-methylene diacrylamide (MBA, AR grade) were used as initiator and cross-linking agent, respectively, and supplied by Xilong Chemical (Shanghai, China). The ethanol, hydrochloric acid, and sodium hydroxide were all analytical grade and used as received.

### 3.2. Preparation of SA-g-(PAA-co-PTM) PASAP

SA (1.125 g) was dissolved in 50 mL distilled water and then added, together with APS at the molar ratio to the total monomer of 5.5 × 10^−3^–9.5 × 10^−3^, into a three-necked flask equipped with a stirring apparatus and a reflux condenser. The reaction mixture was refluxed under the protection of nitrogen at 70 °C in a water bath under stirring for 1 h. MBA and TM with the molar ratios of 0.6 × 10^−2^–1.0 × 10^−2^ and 0–6% to the total monomer, respectively, and the AA neutralized to 20–50% were dissolved in 110 mL deionized water, and added to the flask containing SA and APS solution. The total amount of AA and TM monomers was 0.18 mol. The mixture was stirred for 3 h, and cooled to stop the reaction. The resulting product was washed thoroughly with an ethanol/water mixture (4:1, *v/v*), dried in an oven, and sieved to afford particles with the sizes of 180–425 μm for the swelling and dye adsorption tests. 

### 3.3. Structural Characterization and Swelling Properties Study of SA-g-(PAA-co-PTM) PASAP

#### 3.3.1. FT-IR Spectroscopy

The structures of raw materials and products were conducted on a FTIR spectrometer (NEXUS-470 FTIR, Nicolet Instrument Co., Madison, Wisconsin, USA) using KBr pellets method.

#### 3.3.2. X-ray Diffraction Studies (XRD)

The XRD patterns of the PASAP and SA samples were obtained on the X-ray diffractometer (Rigaku D/Max-1200, Tokyo, Japan) using Ni-filtered Cu Kα radiation at 40 kV and 40 mA (λ = 1.5 Å) and a scanning rate of 0.02°/s. The angle of diffraction varied from 5° to 80°.

#### 3.3.3. Scanning Electron Microscopy (SEM)

The surface morphologies of dried SA and the PASAP were observed with a scanning electron microscope (Hitachi S-4800, Tokyo, Japan). The samples were pre-coated with a ~10 nm thick Au layer using the sputtering technique for SEM imaging.

#### 3.3.4. Thermal Gravimetric Analysis (TGA)

Thermal gravimetric analysis of the samples was made using a thermogravimetric analyzer (DTG-60, Shimadzu Co., Tokyo, Japan) in nitrogen atmosphere at the heating rate of 10 °C/min in the temperature range of 30–500 °C.

#### 3.3.5. Determination of the Swelling Properties of SA-g-(PAA-co-PTM) PASAP

The swelling capacities of the PASAP prepared under different conditions were measured by immersing the polymers in solutions of different pHs or different salt concentrations. Briefly, 0.050 g PASAP was put in a net and immersed in the absorption solution to swell for 90 min. Afterwards, the sample was taken out and the excess liquid on the polymer was removed. The swollen polymer was weighed to calculate its swelling ratio (*Q*, g/g) using Equation (14):

(14) $$Q=\frac{m_{2}-m_{1}}{m_{1}}$$

where *m*_1_ is the weight of the dry polymer (g) and *m*_2_ is the weight of the swollen polymer (g).

### 3.4. Dye Adsorption Properties of SA-g-(PAA-co-PTM) PASAP

The effect of solution pH on the adsorption of MB on the SA-g-(PAA-co-PTM) PASAP with different structures was investigated. Briefly, 0.050 g PASAP was immersed into 50 mL 3000 mg/L MB dye solution at various pHs (adjusted with 1M NaOH or HCl solutions) and allowed to absorb at 303 K for 11 h under constant shaking at 200 rpm. After that, the aqueous phase was collected to determine the residual dye concentration using a UV–VIS spectrophotometer (Pgeneral TU-1810, Beijing Purkinje General Instrument Co., Ltd., Beijing, China).

The dye uptake (mg) per unit mass (g) PASAP at equilibrium (*q_e_*, mg/g) and the adsorption removal% (*R*%) were calculated by Equation (15) and Equation (16), respectively:

(15) $$q_{e}=\frac{\left(c_{0}-c_{e}\right)V}{m}$$

(16) $$R\%=\frac{c_{0}-c_{e}}{c_{e}}\times 100\%$$

where *c*_0_ and *c_e_* are the initial and equilibrium concentrations of the dye solution (mg/L), *V* is the volume of the dye solution (L), and *m* is the mass of the polymer used for the test (g).

#### 3.4.1. Adsorption Kinetics

To investigate the adsorption kinetics of MB on the SA-g-(PAA-co-PTM) PASAP, 0.400 g PASAP was respectively added to 400 mL 2600 mg/L, 2800 mg/L and 3000 mg/L dye solutions in distilled water and agitated on a shaker (200 rpm) at room temperature. One milliliter of dye solution was taken at different time intervals to measure the residual dye concentration as described above with a volume correction.

#### 3.4.2. Adsorption Isotherms

To measure the adsorption isotherms of adsorption of MB on the PASAP, 0.050 g PASAP were immersed in 50 mL aqueous dye solutions of different initial concentrations ranging from 2200 to 3400 mg/L and incubated under 308 K, 318 K and 328 K, respectively, agitated at 200 rpm for 11 h. The residual dye concentrations and the dye uptake per unit mass dry polymer were determined as described above.

#### 3.4.3. Recycling Experiments

Adsorption-desorption experiment cycles were performed to test the reusability of the PASAP. Briefly, 0.050 g PASAP were added to 50 mL 3000 mg/L dye solution in distilled water and agitated (200 rpm) for 11 h. The sample was then filtrated and washed with distilled water to remove the unabsorbed dye and agitated in 200 mL 0.02 M HCl aqueous solution under the same condition. Afterwards, the solid phase of the mixture was filtrated, collected, and thoroughly washed with distilled water for the sake of the next-cycle experiment. The adsorption-desorption cycle was repeated for five times to evaluate the reusability of the SA-g-(PAA-co-PTM) PASAP.

## 4. Conclusions

A new polyampholytic superabsorbent polymer was successfully synthesized via the graft copolymerization of AA and TM monomers onto the SA backbone. Its structure and properties were characterized by FTIR, SEM, XRD, and TGA. The water absorption capacity of the SA-g-(PAA-co-PTM) PASAP synthesized under the optimal conditions reached up to 296.1 g/g. In addition, SA-g-(PAA-co-PTM) PASAP exhibited the typical properties of a polyampholyte, including pH-dependent swelling, high salt-tolerance, and tunable isoelectric point, which endowed it intelligent dye removal ability. Its dye adsorption properties were then investigated using MB as a cationic dye model. The MB adsorption capacity of SA-g-(PAA-co-PTM) PASAP was pH-dependent and decreased with the increase of the relative content of TM. The adsorption kinetic analysis suggests that the MB adsorption obeys the pseudo-second-order kinetics, and both film diffusion and intra-particle diffusion control the MB adsorption. The adsorption isotherm analysis revealed that the adsorption of MB on SA-g-(PAA-co-PTM) PASAP fitted Langmuir isotherm model and, thus, was monolayer. The mean adsorption energy (*E*) and free energy change (Δ*G*°) calculated from D-R isotherm model and adsorption thermodynamics, respectively, indicate that the adsorption is spontaneous chemisorption in nature and enhanced by physical interactions. The MB adsorbed on SA-g-(PAA-co-PTM) PASAP can be desorbed in diluted HCl solution. SA-g-(PAA-co-PTM) PASAP exhibited high MB adsorption efficiency in five adsorption-desorption cycles, showing a good reusability and practicability in MB removal. In conclusion, SA-g-(PAA-co-PTM) PASAP can be potentially used as a high efficiency adsorbent for the dye removal from aqueous solutions. The tunable IEP of SA-g-(PAA-co-PTM) has broadened its application scope in dye adsorption and can simplify the wastewater treatment process.

## Figures and Tables

**Table 1:** Thermogravimetric parameters of SA and SA-g-(PAA-co-PTM) containing 2% TM (TM2%).

Material	Decomposition Step	Temperature Range (°C)	Maximum Decomposition Temperature (°C)	Weight Loss Percentage (%)	Total Weight Loss Percentage (%)	Temperature at 5% Weight Loss (°C)	Temperature at 10% Weight Loss (°C)
SA	1	200–283	251	40.02	40.02	60.20	102.00
SA-g-(PAA-co-PTM)	1	210–300	252	18.91	62.05	158.23	229.67
	2	300–404	371	30.23			
	3	404–477	427	12.91			

**Table 2:** Comparison of the MB removal capacity of different adsorbents.

Adsorbents	Adsorption Capacity (mg/g)	Sources
SA-g-(PAA-co-PTM) (TM2%)	1445.4	Present work
Fox nutshell activated carbon	968.74	[7]
ĸ-carrageenan/graphene oxide gel beads	658.4	[8]
Bamboo-based activated carbon	454.2	[24]
Keratin nanofibrous membranes	170	[25]
Modified Ball clay	100	[26]
Chitosan/sepiolite composite	40.986	[27]

**Table:** **a.** Adsorption kinetic parameters of MB on SA-g-(PAA-co-PTM) PASAP (TM2%).

Models	Parameters	Dye Concentrations(mg/L)		
		2600	2800	3000
Pseudo-first-order model	$k_{1}$ (min^−1^)	2.508	6.866	3.944
	$R_{1}^{2}$	0.9814	0.9851	0.9428
Pseudo-second-order model	$k_{2}$ (g/(mg min))	1.00 × 10^−4^	5.86 × 10^−5^	4.04 × 10^−5^
	$R_{2}^{2}$	0.9999	0.9999	0.9997
Weber Morris model	$k_{P1}$ (mg^2^/(g^2^ min))	7.00 × 10^4^	1.01 × 10^5^	7.94 × 10^4^
	$k_{P2}$ (mg^2^/(g^2^ min))	3.24 × 10^3^	6.85 × 10^3^	1.04 × 10^4^
	$k_{P3}$ (mg^2^/(g^2^ min))	6.53 × 10^2^	9.85 × 10^2^	1.79 × 10^3^
	C	4.83 × 10^6^	4.70 × 10^6^	4.68 × 10^6^
	$R_{p2}^{2}$	0.8333	0.8222	0.9527
Boyd model	$k_{fd}$ (min^−1^)	0.043	0.043	0.027
	$R^{2}$	0.9470	0.9299	0.9574

**Table:** **b.** Adsorption isotherm parameters of MB on SA-g-(PAA-co-PTM) PASAP (TM2%).

Model	Parameters	308 K	318 K	328 K
Langmuir	*K_L_* (L/mg)	0.0236	0.0208	0.0205
	*R* ^2^	0.9976	0.9994	0.9990
	*R_L_*	0.0208	0.0235	0.0238
Freundlich	*K_F_* (mg/g)	1205.4	1098.9	1182.6
	*b_F_*	0.126	0.135	0.114
	*R* ^2^	0.9728	0.9548	0.9744
Dubinin-Radushkevich	*K* (J^2^/mol^2^)	−2.015 × 10^−4^	−6.973 × 10^−4^	−4.743 × 10^−4^
	*R* ^2^	0.5941	0.8802	0.6788
	*E* (kJ/mol)	49.81	26.78	32.47

**Table 4:** Adsorption thermodynamic parameters of MB on SA-g-(PAA-co-PTM) PASAP (TM2%).

*T*(K)	Δ*G*° (kJ/mol)	*T*Δ*S*° (kJ/mol)	Δ*H*° (kJ/mol)	Δ*S*° (J/(mol K))
308	−23.75	17.69	−6.02	57.44
318	−24.19	18.27		
328	−24.91	18.84		
